# A Highly Sensitive Graphene-Based Terahertz Perfect Absorber Featuring Five Tunable Absorption Peaks

**DOI:** 10.3390/ma18112601

**Published:** 2025-06-03

**Authors:** Hongyu Ma, Pengcheng Shi, Zao Yi

**Affiliations:** 1 Joint Laboratory for Extreme Conditions Matter Properties, Key Laboratory of Manufacturing Process Testing Technology of Ministry of Education, State Key Laboratory of Environment-Friendly Energy Materials, Southwest University of Science and Technology, Mianyang 621010, China; 15082734560@163.com (H.M.); pengchengshi114@163.com (P.S.); 2 School of Chemistry and Chemical Engineering, Jishou University, Jishou 416000, China

**Keywords:** graphene, THz, narrow band, highly sensitive, tunable absorber, perfect absorber in multi-band

## Abstract

In this article, we present a high-sensitivity narrow-band perfect graphene absorber that exhibits excellent tunability across multiple bands. The top layer of the absorber unit is composed of graphene material, and the shape is a square graphene layer with a ring structure and a square structure removed from the middle. A SiO_2_ dielectric layer is located in the middle, and a layer of gold substrate exists at the bottom. This structure has generated five perfect absorption peaks at 6.08216 THz, 7.29058 THz, 9.34669 THz, 11.5471 THz, and 13.0441 THz, and the levels of absorption are 98.24%, 98.03%, 99.55%, 98.87%, and 99.99%, respectively. We have proved the advantages of our model by comparing the influence of different shapes of graphene on the absorption rate of the model. Then, we changed the relaxation time and Fermi energy level of graphene and other factors such as the refractive index to prove that our structure has good tunable performance. Finally, we calculated the sensitivity, and the sensitivity of this structure is as high as 4508.75 GHZ/RIU. Compared with previous articles, our article has more absorption peaks, a higher absorption efficiency, and a higher sensitivity. The absorber proposed in this paper shows great potential to contribute to high-sensitivity sensors, photoelectric detection, photoelectric communication, and other related fields.

## 1. Introduction

In recent years, light absorption technology has become an extremely popular area of scientific research, with applications in various fields such as solar absorbers, infrared detection, solar thermal emitters, solar cells, and photosensors [1,2,3]. Narrow-band absorption technology with high sensitivity is widely used in high-sensitivity sensors [4,5,6]. Precisely because of its significant research value and broad application areas, more and more scholars and teams have recently employed various methods to attempt to improve light absorption efficiency [7,8]. A steady stream of novel devices has emerged, including broad-band and narrow-band absorbers, single- and multi-frequency absorbers, metasurface absorbers, tunable absorbers, and so on [9,10,11]. All of these have made significant contributions to the development of light absorption technology.

Terahertz (THz) refers to a unique electromagnetic wave with a frequency range of 0.1–10 THz and a wavelength range of 30–3000 μm, falling between microwaves and infrared waves [12,13,14]. Terahertz waves, which are part of the electromagnetic spectrum between microwaves and infrared waves, possess unique physical properties. They can penetrate certain materials similar to microwaves and exhibit refraction and reflection characteristics like other light waves [15,16]. Terahertz waves were officially named in the mid-to-late 1980s. Terahertz has had a significant impact on research in various fields and holds broad application prospects in areas such as optical communications, biological imaging, and security inspection [17,18]. For instance, terahertz imaging technology can be used for non-contact scanning detection, enabling high-speed and accurate three-dimensional scanning of the objects to be inspected [19]. Terahertz communication technology, due to its high speed, large capacity, and strong anti-interference capabilities, has become an important research and development direction for future wireless communication technologies. Further research into terahertz and its applications in the terahertz band holds significant importance for advancing human technology and improving living standards.

Graphene is an allotrope of carbon, a novel two-dimensional nanomaterial with a single-layer hexagonal honeycomb lattice structure. Graphene boasts a large surface area, high hardness, and mechanical strength. Simultaneously, it exhibits excellent flexibility [20,21,22]. Furthermore, graphene exhibits remarkable thermal and electrical conductivity. In thermoelectric applications, this property is highly valuable. For example, in the fabrication of thermoelectric generators, graphene can potentially enhance the conversion efficiency between heat and electricity. Its high electrical conductivity allows for efficient charge transport, while the good thermal conductivity helps in the heat transfer process, which is crucial for thermoelectric performance [23,24,25]. Thanks to these virtues, graphene finds widespread application in various fields, including electronics, materials science, energy, medical, and environmental fields. In the energy field, graphene can be used to fabricate high-efficiency batteries, capacitors, and absorbers [26,27,28]. Graphene materials absorb little light in the natural environment. In recent years, with the deepening of research, many absorbers based on graphene materials have been developed, such as narrow-band absorbers, broad-band absorbers, and terahertz wave absorbers, greatly enhancing the absorption efficiency of graphene [29,30,31]. In some structures, the absorption rate of graphene even approaches 100%.

In this study, we have designed a graphene-based metamaterial perfect absorber. The uppermost layer of the absorber consists of a graphene structure having a circular ring and a square structure excavated from the middle. Within this structure, the graphene layer generates five perfect absorption peaks and one tunable absorption peak in the 5–14 THz range. This paper primarily explores the absorption regulation and application of the first five absorption peaks, and further details will not be elaborated in subsequent sections. The absorption rates corresponding to these five perfect absorption peaks are 98.24%, 98.03%, 99.55%, 98.87%, and 99.99%, demonstrating ultra-high absorption efficiency. Through the analysis of electric field diagrams in this article, we gained an understanding of the causes of several absorption peaks. In addition, because of the excellent tunable properties of graphene materials, we verified the good tunability of the absorber structure by adjusting the relaxation time of graphene and different Fermi levels. Additionally, by altering several structural parameters of the top-layer graphene structure of the absorber, we demonstrated the structural advantages of this absorber. Finally, to confirm the practical application value of the absorber, we compared and calculated its sensitivity. The results showed that our absorbing device exhibits high sensitivity and possesses considerable practical application capabilities and value.

## 2. Numerical Model and Structural Design

**Figure 1:**
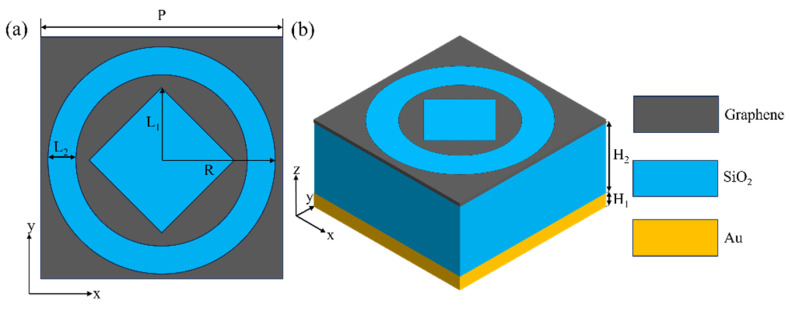
(**a**) Diagram of the absorber unit structure. (**b**) Structure and parameters of the xy plane under the unit structure. Among them, the thickness of the graphene used in the top layer is 1 nm.

The absorber structure proposed in this article is illustrated in Figure 1, consisting of a classic three-layer configuration. Starting from the top, the topmost layer is composed of graphene material, followed by a silica (SiO_2_) dielectric layer in the middle and a 300 nm gold substrate at the bottom. The graphene layer is especially remarkable because of its rather ingenious design concept. The inspiration for the initial structure of the top-layer graphene came from ancient Chinese coins. The circular–square combination in ancient coins has unique electromagnetic features. This symmetry can create special electromagnetic field distributions when interacting with terahertz waves. The ring and square-cut parts act as resonant elements. Their edges concentrate the electric field. Based on the localized surface plasmon resonance (LSPR) principle, resonance enhances terahertz absorption. Preliminary simulations show that this structure has more absorption peaks than simple ones. After that, we optimized the parameters to determine the best structure. Through continuous parameter scanning for parameter optimization, we analyzed and calculated the dynamic changes in the absorption effect under the different structural parameters of the device and, finally, determined this set of optimal structural parameters [32,33]. Some of the more important analytical images and results of the parameter scans will be presented later in the article. Moreover, it has a unique and novel pattern: a ring and a square are cut out from a 1 nm thick graphene sheet. The diagonal length of the excised square measures 6 μm, with L_1_ equal to 3 μm. The outer and inner diameters of the excised circular ring are R = 4.7 μm and 3.5 μm, respectively, resulting in L_2_ = 1.2 μm. The pitch is set at P = 10 μm. The SiO_2_ layer has a thickness of H_2_ = 5 μm, and the refractive index of SiO_2_ is n = 1.4. Finally, the thickness of the gold substrate, H_1_, is equal to 0.3 μm. The dielectric constant of the gold substrate is obtained through the Drude model [34]. The simplicity of the structure and the ease of obtaining the materials make this graphene absorber highly suitable for manufacturing. In order to have a more intuitive understanding of the specific parameters of the device, we have created Table 1 to directly present the parameter values of the absorption device.

**Figure 2:**
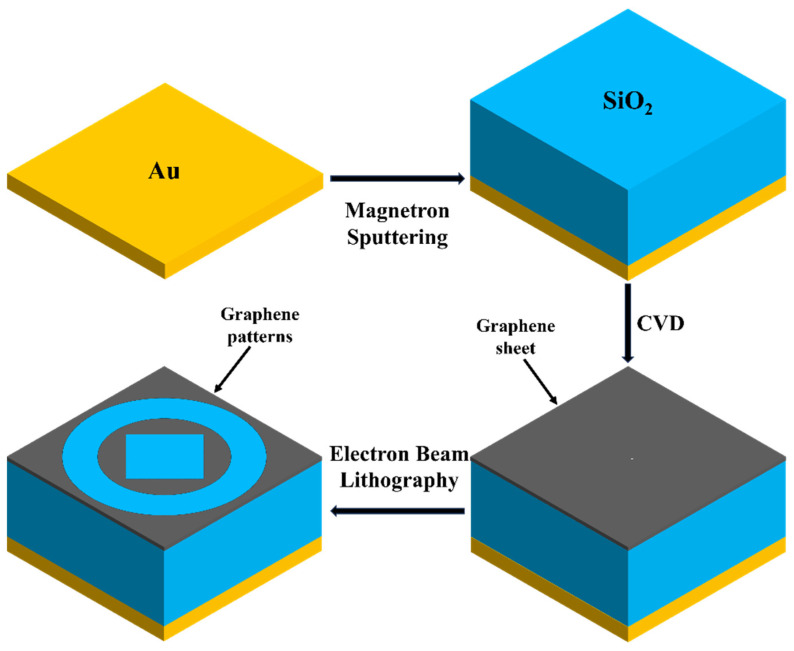
The manufacturing flowchart of the graphene absorber proposed in this article.

As shown in Figure 2, in the actual manufacturing process, using current technological capabilities, first, a 5-micrometer-thick silicon dioxide (SiO_2_) film can be deposited on a gold (Au) substrate using magnetron sputtering. Then, graphene sheets are independently prepared via chemical vapor deposition (CVD) and transferred onto the SiO_2_ dielectric layer. Finally, electron beam lithography can be easily employed to obtain the desired graphene patterns. This is a simplified fabrication process for the device [35,36].

The Drude model formula for Au described above is as follows:

(1) $$\varepsilon_{Au}=9-\frac{\omega_{p}^{2}}{\omega_{p}^{2}+i\omega \gamma }$$

In the above formula, i is the unit of the imaginary part, and ω represents the angular frequency, with a damping constant γ = 1.23 × 1014 s^−1^ and a plasma frequency $\omega_{p}$ = 1.37 × 1016 s^−1^ [37,38].

The formula for the surface conductivity of monolayer graphene is as follows [39]:

(2) $$\sigma \left(\omega \right)=\frac{e^{2}E_{f}}{\pi h^{2}}\frac{i}{w+i\tau^{-1}}$$

In the above formula, $E_{f}$ depends on the charge concentration. In the research of this paper, the thickness of the graphene we used is one nanometer, with a Fermi level $E_{f}$ = 1.00 ev. The Fermi level of graphene can be altered through chemical doping [40].

We used the finite-difference time-domain (FDTD) simulation method to simulate and calculate the proposed model and, specifically, used the Lumerical FDTD software to obtain the simulation and calculation results. For the boundary conditions in the simulation process, they can be easily set in the software [41,42,43]. We set perfectly matched layers in the *Z*-axis direction and simultaneously used periodic boundary conditions on the *X*-axis and *Y*-axis planes. In this way, we can analyze the properties of the device throughout the entire period by simulating a unit structure of the device. We used electromagnetic waves with a frequency range of 5–14 THz to vertically incident on the top-layer graphene structure of the device, so as to explore the narrow-band absorption characteristics of the device. The absorption rate of the absorption device can be obtained by the formula A = 1 − R − T [44,45,46]. Because we used a Au substrate, and its thickness is significantly greater than the skin depth of Au within this frequency range, which means that the substrate can achieve total reflection of the light and avoid the occurrence of transmission, the calculation formula for the absorption rate can also be simplified to A = 1 − R.

## 3. Results and Discussion

**Figure 3:**
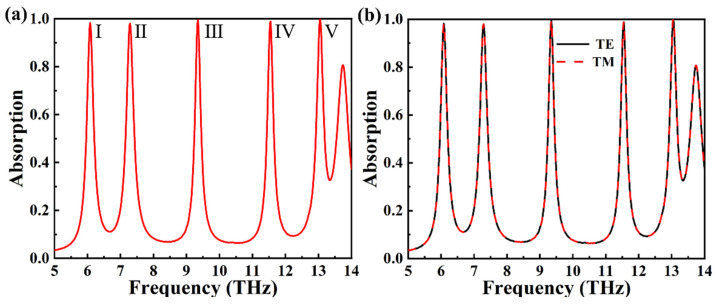
(**a**) The absorption spectrum diagram of the graphene absorber proposed in this paper. (**b**) Absorption spectra of TM mode transformed to TE mode (polarization angle 0–90°).

Figure 3a displays the absorption spectrum of the graphene absorber structure described in this paper. As can be seen from the figure, our absorber reaches maximum absorption rates of 98.24%, 98.03%, 99.55%, 98.87%, and 99.99% at 6.08216 THz, 7.29058 THz, 9.34669 THz, 11.5471 THz, and 13.0441 THz, respectively. In subsequent descriptions, we will refer to the five absorption peaks mentioned above as I, II, III, IV, and V. Figure 3b demonstrates the variation in the absorption spectrum of this structure under TE and TM waves. As can be observed from the figure, the absorption spectra for TE and TM waves nearly overlap, demonstrating the excellent polarization insensitivity of this absorber structure.

**Figure 4:**
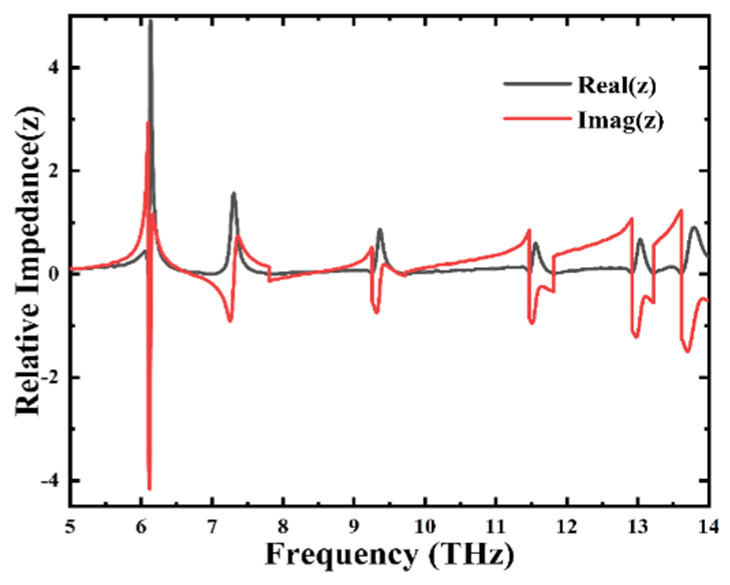
The presented figure illustrates the relative impedance of the graphene absorber, wherein the imaginary part of the impedance is denoted by the red solid line, while the real part is indicated by the black solid line.

To precisely investigate the origin of the absorber’s absorption peaks and illustrate the mechanism of achieving perfect absorption, we applied the principle of effective impedance matching using an ideal electromagnetic metamaterial absorber to calculate the relative impedance Z. Specifically, as illustrated in Figure 4, this figure depicts the variation relationship between the incident frequency and the impedance of the five absorption peaks of the graphene absorber. When the absorber reaches the critical coupling condition (i.e., when the equivalent impedance of the absorber is consistent with the free-space impedance), the relative impedance Z equals one [47,48]. At this moment, the absorption device exhibits strong electromagnetic absorption characteristics, which signifies the occurrence of perfect absorption. The expression for the aforementioned relative impedance Z is as follows [49,50]:

(3) $$Z=\pm \sqrt{\frac{{\left(1+S_{11}\right)}^{2}-S_{21}^{2}}{{\left(1-S_{11}\right)}^{2}-S_{21}^{2}}}$$

In Formula (3), $S_{11}$ and $S_{21}$ indicate the reflection and transmission coefficients, respectively. In Figure 4, the real part and the imaginary part of the absorber’s impedance are represented by black and red, respectively. Combining the image data with the existing formulas, at the five absorption peaks, we observe that the real part of the relative impedance of the proposed absorber is close to 1, while the imaginary part is close to 0. This means that the reflection of the system is suppressed, confirming the emergence of perfect absorption peaks and indicating that the device conforms to the impedance matching theory.

**Figure 5:**
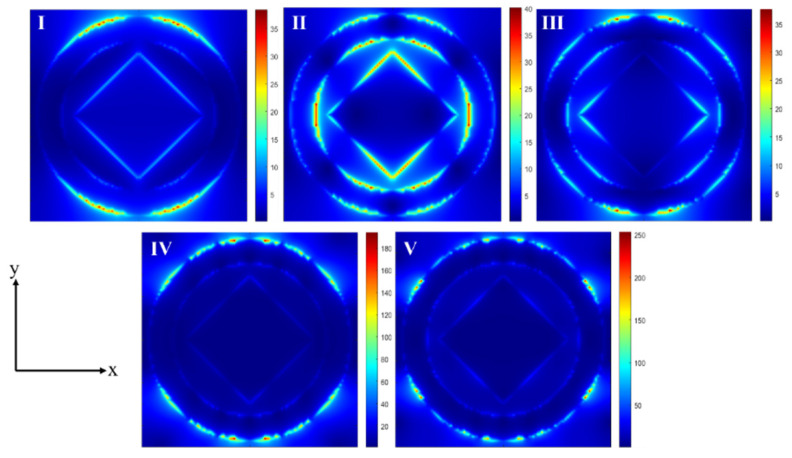
The five absorption peaks, labeled I, II, III, IV, and V, correspond to the diagrams of the electric field of the graphene design in the xy direction at frequencies of 6.08216 THz, 7.29058 THz, 9.34669 THz, 11.5471 THz, and 13.0441 THz, respectively.

To systematically analyze the physical origin of the absorption peaks in the designed absorption model, this study conducts a quantitative analysis on the electric field distributions in the xy plane at the characteristic peak values of absorption bands I, II, III, IV, and V, with relevant results shown in Figure 5. Based on the theoretical framework of localized surface plasmon resonance (LSPR) [51,52], this research focuses on the resonance mechanism of electromagnetic wave–micro-/nano-structure interactions. When electromagnetic radiation with specific photon frequencies acts on the absorber surface, if the incident wave frequency matches the intrinsic resonance frequency of the surface plasmons in the structure, then a localized surface plasmon resonance (LSPR) mode will be excited, leading to the highly localized distribution of electromagnetic field energy on the micro-/nano-structure surface and significantly enhancing the photon energy coupling efficiency [53]. It is worth noting that Dakhlaoui et al. confirmed via the transfer matrix method that resonant states in graphene modulated by multi-electrostatic barriers can arise from solving transport-like Schrödinger equations, with the transmission coefficient showing multi-peak characteristics as the number of barriers increases. Zhan et al. theoretically demonstrated that graphene plasmon resonances can be regulated by structural parameters and that interference effects in multi-layer graphene can generate photonic bandgaps [54,55]. These theoretical methods and analytical approaches also provide important references for exploring the resonance mechanisms of similar structures.

At the characteristic frequency of 6.08216 THz, the effective interaction region of surface plasmon resonance is primarily concentrated on the edge ridges of the central hollow square structure and the vertical sidewalls of the outer ring in the peripheral annular hollow structure. For the frequency point of 7.29058 THz, the electric field intensity distribution nephogram reveals the presence of cross-scale electromagnetic coupling effects, which increases the proportion of energy leakage into the radiation field and, consequently, causes a decline in the overall absorption efficiency of the structure. Combining the frequency-domain response characteristics of LSPR theory, this phenomenon can be attributed to the dominant role of radiation damping effects in the plasmon mode at this frequency, thus explaining the physical essence of the lower absorption efficiency of the graphene layer at absorption peak II compared to the other characteristic peaks [56]. The electromagnetic field energy at 9.34669 THz exhibits significant spatial localization characteristics, mainly enriched in the edge regions of the inner–outer ring structural interface and the left–right corner areas of the central square structure. Compared with the frequency points of 6.08216 THz and 11.5471 THz, the electromagnetic field localization region at this frequency has a larger mode volume, leading to a significant enhancement of light–matter interaction intensity at absorption peak III and, thus, demonstrating higher light absorption efficiency. At the characteristic frequencies of 11.5471 THz and 13.0441 THz, the electromagnetic field distribution presents an outer-ring structure-dominated local resonance mode, with energy primarily confined near the outer side walls of the peripheral annular hollow structure. From the perspective of energy transmission and loss mechanisms, the high-efficiency wave absorption characteristics of this absorber structure result from the synergistic effect of multiple physical phenomena: Firstly, the LSPR effect induced on the graphene surface by terahertz-band electromagnetic waves significantly enhances light–matter interaction efficiency through the near-field localization of electromagnetic field energy [57]. Secondly, the total reflection property of the gold substrate causes incident electromagnetic waves to form multi-path interference at the graphene–dielectric–metal interface, enhancing energy deposition efficiency through multiple reflection–absorption cycles. The synergistic effect of these mechanisms provides theoretical support and structural paradigms for designing terahertz functional devices with wide bandwidth and high absorption efficiency.

**Figure 6:**
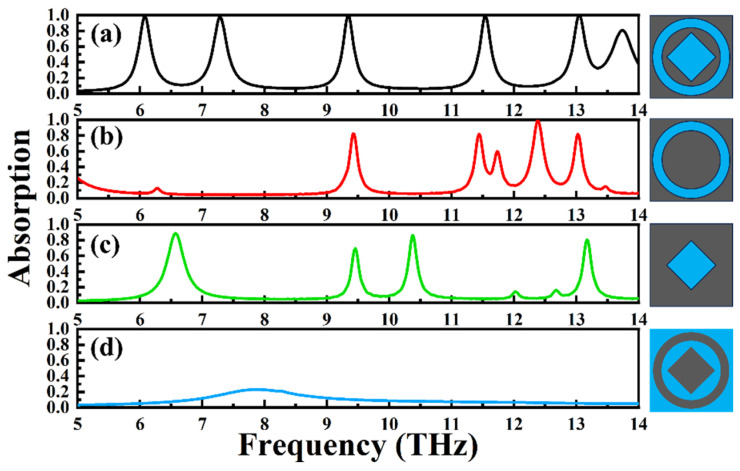
(**a**) The absorption spectrum of the graphene pattern put forward in this paper. (**b**) The absorption spectrum of the graphene layer with only one circular ring structure removed, shown by a solid red line. (**c**) The absorption spectrum of the graphene layer with only one square structure removed, shown by a solid green line. (**d**) The absorption spectrum of the pattern consisting of one circular ring of graphene and square graphene, shown by a solid blue line.

In Figure 6, we modified the graphene pattern on the top layer to demonstrate the benefits of our absorber. We altered the graphene structure to include scenarios where only one circular ring is removed from the graphene layer, where only one square is removed, and where the graphene pattern consists of just one circular ring and one square. As we have previously explained the absorption characteristics of the structure in Figure 6a, we will not discuss them again here. In Figure 6b, the absorption spectrum of the graphene changes from the five absorption peaks observed in our structure to only three peaks, with only one peak reaching an absorption rate above 90%. This is significantly inferior to the overall absorption rate of our proposed structure. In Figure 6c, the absorption spectrum of the graphene transforms from five peaks in our structure to four peaks, with all four peaks having absorption rates below 90%. Again, the overall absorption rate is far worse than that of our structure. Finally, in Figure 6d, there are no absorption peaks in the graphene absorption spectrum, which is clearly inferior to our proposed graphene structure. In summary, by altering the top-layer graphene pattern, we have demonstrated the superiority of our proposed structure.

**Figure 7:**
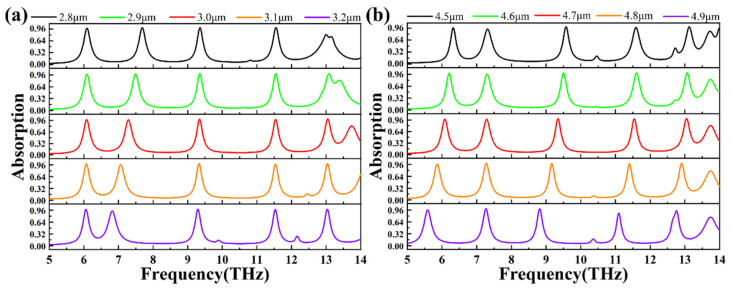
(**a**) The absorption spectra diagram under changes of the graphene layer parameter L_1_ of sizes 2.8 μm, 2.9 μm, 3.0 μm, 3.1 μm, and 3.2 μm. The spectra are represented by black, green, red, orange, and purple, respectively. (**b**) Absorption spectra of graphene with graphene parameter R of sizes 4.5 μm, 4.6 μm, 4.7 μm, 4.8 μm, and 4.9 μm. Similarly, the spectra are denoted by black, green, red, orange, and purple, respectively.

In the study mentioned above, we discovered that the absorption peaks of our proposed structure are primarily generated by the circular and square structures excised from the graphene layer. Consequently, we altered the sizes of these excised circular and square structures in the graphene layer to further investigate the advantages and tunable performance of our absorber. As depicted in Figure 7a, we varied the size of L_2_ consecutively to 2.8 μm, 2.9 μm, 3.0 μm, 3.1 μm, and 3.2 μm, plotting the corresponding graphene absorption spectra accordingly. From the graph, it is evident that the overall absorption rates of peaks I, II, III, IV, and V remain relatively unchanged. By increasing L_2_ from 2.8 μm to 3.2 μm, peak II shifts from 7.68737 THz to 6.82164 THz, exhibiting a regular blue shift, while peak III also experiences a slight blue shift from 9.34669 THz to 9.29259 THz. 

Immediately following, in Figure 7b, we varied the size of L_1_ consecutively to 4.5 μm, 4.6 μm, 4.7 μm, 4.8 μm, and 4.9 μm, plotting the corresponding graphene absorption spectra accordingly. As L_1_ changes from 4.5 μm to 4.9 μm, peak I shifts from 6.31663 THz to 5.57715 THz, peak II shifts from 7.30862 THz to 7.27255 THz, peak III shifts from 9.58116 THz to 8.80561 THz, peak IV shifts from 11.5832 THz to 11.0962 THz, and peak V shifts from 13.1162 THz to 12.7555 THz. Overall, the five absorption peaks of the absorber exhibit blue shifts of varying magnitudes, with peak II experiencing a relatively smaller blue shift. Regarding absorption rates, comparing the L_1_ values of 4.5 μm and 4.7 μm, the absorption rates of peaks I, II, IV, and V are lower for 4.5 μm, while the absorption rate of peak III is slightly higher at 99.76% for 4.5 μm compared to 99.55% for 4.7 μm. Similarly, comparing 4.6 μm and 4.7 μm, the absorption rates of peaks II, III, IV, and V are lower for 4.6 μm, with the peak I absorption rate slightly higher at 98.85% for 4.6 μm compared to 98.24% for 4.7 μm. When comparing 4.7 μm and 4.8 μm, only the absorption rate of peak II is slightly higher at 99.44% for 4.8 μm compared to 98.03% for 4.7 μm, with the absorption rates of other peaks being lower for 4.8 μm. Lastly, comparing the L_1_ values of 4.7 μm and 4.9 μm, only the absorption rate of peak II is slightly higher at 99.42% for 4.9 μm compared to 98.03% for 4.7 μm, with the absorption rates of other peaks being lower for 4.9 μm. In summary, our absorber can be tuned by altering the structural parameters of L_1_ and L_2_, achieving optimal overall absorption rates at L_1_ = 4.7 μm and L_2_ = 3.0 μm.

To further explore the properties and advantages of this absorber structure, we will keep the original structural parameters unchanged and alter the Fermi level and relaxation time of graphene. The Fermi level of graphene can be manipulated by adjusting the external voltage. Specifically, a simple circuit path can be formed by applying an external bias voltage to the device. A layer of ionic gel conductive layer is coated on the top of the device, and the Au substrate is used as the other part of the conductive layer. And the specific formula is given below [58,59]:

(4) $$E_{f}=V_{f}\sqrt{\frac{\pi \varepsilon_{0}\varepsilon_{\gamma }V_{0}}{e_{0}n_{s}}}$$

(5) $$\tau =\frac{\mu E_{f}}{e_{0}V_{i}^{2}}$$

In Formula (4), $V_{f}$ represents the Fermi velocity, $V_{0}$ represents the magnitude of the external voltage, $e_{0}$ refers to the number of electron charges, and $n_{s}$ is used to represent the number of layers of SiO_2_ in the structure. In Formula (5), $\mu =10^{4}\;{cm}^{2}/(V.S)$ represents the carrier mobility, $E_{f}$ is the Fermi level in Formula (4), and $V_{i}=10^{6}\;m/s$ is the Fermi velocity mentioned above.

**Figure 8:**
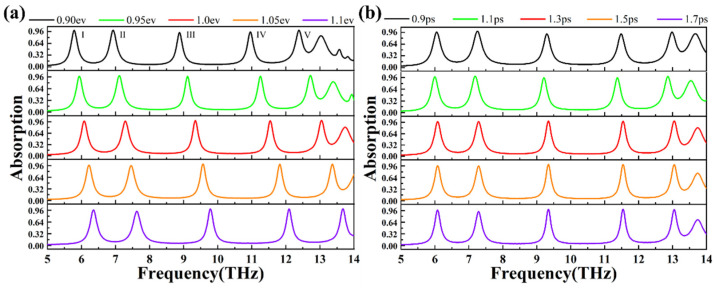
(**a**) Absorption spectra associated with graphene having Fermi levels of 0.90 ev, 0.95 ev, 1.0 ev, 1.05 ev, and 1.1 ev. We make use of solid black, green, red, orange, and purple lines to signify the absorption spectra for 0.90 ev, 0.95 ev, 1.0 ev, 1.05 ev, and 1.1 ev, respectively. (**b**) Absorption spectra of graphene with relaxation times of 0.9 ps, 1.1 ps, 1.3 ps, 1.5 ps, and 1.7 ps. We make use of solid black, green, red, orange, and purple lines to signify the absorption spectra for 0.9 ps, 1.1 ps, 1.3 ps, 1.5 ps, and 1.7 ps, respectively.

As shown in Figure 8a, we investigated the change in the light absorption of graphene layers as the Fermi level varied from 0.90 ev to 1.1 ev. As the Fermi level went up from 0.90 ev to 1.1 ev, peak I shifted from 5.77555 THz to 6.35271 THz, peak II shifted from 6.92986 THz to 7.61523 THz, peak III shifted from 8.87776 THz to 9.77956 THz, peak IV shifted from 10.9519 THz to 12.0882 THz, and peak V shifted from 12.3768 THz to 13.6754 THz. From the perspective of absorption rates, when the graphene Fermi level went up from 0.9 ev to 0.95 ev, the rate of absorption of peak I decreased from 99.29% to 98.22%, the absorption rate of peak II decreased from 99.97% to 99.49%, the rate of absorption of peak III went up from 93.86% to 97.59%, the rate of absorption of peak IV went up from 95.39% to 97.58%, and the rate of absorption of peak V went up from 99.21% to 99.99%. As the graphene Fermi level went up from 0.95 ev to 1.0 ev, the rate of absorption of peak I went up from 98.22% to 98.24%, the rate of absorption of peak II decreased from 99.49% to 98.03%, the rate of absorption of peak III went up from 97.59% to 99.55%, the rate of absorption of peak IV went up from 97.58% to 98.87%, and the rate of absorption of peak V remained at 99.99%. When the graphene Fermi level went up from 1.0 ev to 1.05 ev, the rate of absorption of peak I decreased from 98.24% to 97.02%, the rate of absorption of peak II decreased from 98.03% to 95.96%, the rate of absorption of peak III decreased from 99.55% to 99.40%, the rate of absorption of peak IV went up from 98.87% to 99.87%, and the rate of absorption of peak V remained at 99.99%. Finally, as the graphene Fermi level went up from 1.05 ev to 1.1 ev, the absorption rate of peak I decreased from 97.02% to 97.01%, the rate of absorption of peak II decreased from 95.96% to 92.97%, the rate of absorption of peak III went up from 99.40% to 99.70%, the rate of absorption of peak IV decreased from 99.87% to 99.62%, and the rate of absorption of peak V decreased from 99.99% to 99.97%. Taken together, our absorber can be effectively tuned by adjusting the Fermi level. Most peaks maintain absorption rates above 95%, and the overall absorption efficiency is optimal at a Fermi level of 1.0 ev, with absorption rates exceeding 98% for all five peaks.

Indeed, we set the Fermi level of graphene within the absorber to be 1.0 ev, as well as observed changes in the absorber’s absorption rate by adjusting the relaxation time of graphene from 0.9 ps to 1.7 ps, as shown in Figure 8b. As the relaxation time increased from 0.9 ps to 1.1 ps, the absorption rate at peak I rose from 96.72% to 98.03%, peak II from 99.44% to 99.84%, peak III from 91.88% to 95.16%, peak IV from 91.61% to 95.33%, and peak V from 96.66% to 99.09%. When the relaxation time increased from 1.1 ps to 1.3 ps, the peak I absorption increased from 98.03% to 98.24%, peak II decreased from 99.84% to 98.03%, peak III rose from 95.16% to 99.55%, peak IV from 95.33% to 98.87%, and peak V from 99.09% to 99.99%. As the relaxation time increased from 1.3 ps to 1.5 ps, peak I absorption decreased from 98.24% to 96.99%, peak II from 98.03% to 95.96%, peak III from 99.55% to 99.49%, peak IV increased from 98.87% to 99.78%, and peak V from 99.99% to 99.71%. Finally, as the relaxation time increased from 1.5 ps to 1.7 ps, the peak I absorption rose from 96.99% to 97.06%, peak II decreased from 95.96% to 93.29%, peak III from 99.49% to 99.58%, peak IV from 99.78% to 99.44%, and peak V from 99.71% to 98.44%. Based on these absorption data, it is evident that the overall absorption rate is optimal when the relaxation time is 1.3 ps, with all five peaks achieving absorption rates above 98%. Although the absorption rates fluctuate somewhat at different relaxation times, the overall absorption rates remain above 91%. Thus, we hypothesize that the relaxation time will have an impact on the overall absorption rate of the absorber, and if the relaxation time is brief, the surface plasmon resonance of graphene will not be conducive to being stimulated, so the absorption rate of some absorption peaks will decrease [60,61]. When the relaxation time is long, the surface plasmon oscillation of graphene approaches saturation. Because that part of the light is reflected, the absorption rate of some peak values will decrease.

Sensitivity (S) is one of the main indicators reflecting the absorption performance of an absorber, so we will use sensitivity to describe the absorber we present. In addition, we also calculated the quality factor (Q) and the figure of merit (FOM) value of the absorber [62,63]. The corresponding formula is expressed as follows [64,65]:

(6) $$S=\frac{\Delta \delta }{\Delta n}$$

(7) $$Q=\frac{\lambda }{FWHM}$$

(8) $$FOM=\frac{S}{FWHM}$$

In Formula (5), S represents sensitivity, Δδ refers to the shift in absorption peak frequency due to changes in the index of refraction of the environment, and Δn represents the change in the index of refraction of the environment. In Formulas (6) and (7), the term “FWHM”, which stands for “full width at half maximum”, is commonly utilized to characterize the width corresponding to the point where the height of a peak is reduced to half its maximum value, such as in the context of spectral domains and peak curves.

**Figure 9:**
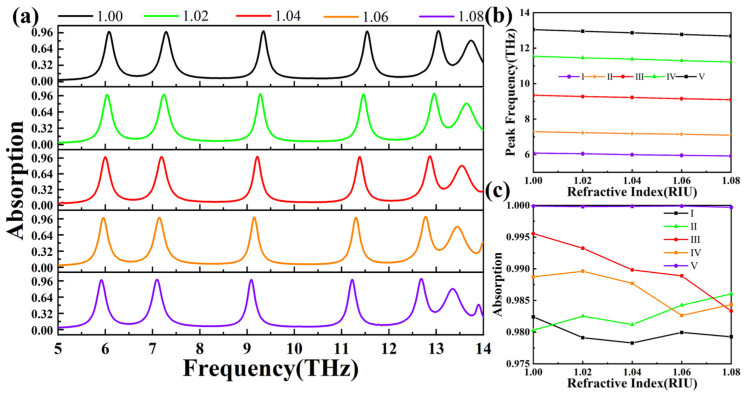
(**a**) Alterations in the absorption spectrum of the absorber as the environmental refractive index increases from 1 to 1.08. (**b**) Images showing the frequency variations of the five absorption peaks as the environmental refractive index goes up from 1 to 1.08. (**c**) Variations in the absorption indices of the five absorption peaks as the environmental refractive index increases from 1 to 1.08. In this figure, solid lines of black, green, red, orange, and purple are used to serve as the representation of absorption peaks I, II, III, IV, and V, respectively.

In order to facilitate the determination of the absorptive sensitivity and continue to deeply explore the adjustable mechanism of the absorber we have presented, we have drawn the absorption spectrum of graphene under different environmental refractive indices, and then through specific analysis and processing and by using the sensitivity formula mentioned in the previous text, the sensitivity of the absorber can be calculated [66,67,68]. As shown in Figure 9a, our absorber exhibits distinct graphene absorption spectra under varying refractive indices of the external environment. Using this data, we derived Figure 9b, which allows us to quickly determine the sensitivity of our absorber. The sensitivities of peaks I, II, III, IV, and V are 2029 GHZ/RIU, 2408 GHZ/RIU, 3156.25 GHZ/RIU, 4058.75 GHZ/RIU, and 4508.75 GHZ/RIU, respectively. On the premise that the sensitivity and absorption parameters are known, with the help of Formulas 6 and 7 mentioned above, it is not difficult for us to calculate the quality factor and FOM corresponding to the five absorption peaks. Among them, Q_I_ = 25.34, Q_II_ = 27.00, Q_III_ = 42.48, Q_IV_ = 51.32, Q_V_ = 47.43, FOM_I_ = 8.45 1/RIU, FOM_II_ = 10.9 1/RIU, FOM_III_ = 14.35 1/RIU, FOM_IV_ = 18.03 1/RIU, and FOM_V_ = 16.39 1/RIU. Figure 9c illustrates the changes in absorption rates of each absorption peak as the refractive index of the environment varies. It is evident from the figure that despite changes in the refractive index, the absorption rates of our absorber’s five peaks remain largely unchanged, all exceeding 97.55%, demonstrating its exceptional absorption efficiency.

In Table 2 [69,70,71,72,73,74,75], we compare the absorber presented in this paper with those reported in previous articles. When comparing with the four articles that also use graphene materials in the table, we can observe that the absorber we developed has more absorption peaks than three of them. Moreover, while achieving multiple absorption peaks, its overall absorption effect is also very excellent. In addition, this device is far superior to other graphene absorbers in terms of both the sensitivity quality factor and the q value. Compared with the three articles using Au materials and BDS, our absorber also has more absorption peaks, a higher absorption rate, higher sensitivity, and a better quality factor. The various comparison results shown in the table strongly indicate that graphene has a good prospect in the application of metamaterial absorbers. An absorber with high absorption, high sensitivity, a high quality factor, and a high Q value, like the one in this paper, will have broader applications and development space in fields such as high-sensitivity sensors, photoelectric detection, and optical communication.

## 4. Conclusions

This paper presents a narrow-band absorber with five tunable, high-sensitivity perfect absorption peaks in the terahertz range. The absorber is composed of a classical metamaterial absorber structure, consisting of a gold substrate at the bottom, a SiO_2_ dielectric layer in the middle, and a top layer made up of a graphene sheet with a circular ring and a square structure removed from the center. We explain the mechanism behind the absorber’s absorption peaks using electric field principles. Subsequently, we demonstrate the advantages of our proposed structure by altering the graphene pattern on the top layer. Furthermore, we show that the absorber exhibits excellent tunability by adjusting various parameters such as the structural parameters of the graphene layer, the Fermi level, and the relaxation time of the graphene. This allows for the frequency of the absorption peaks to be altered through the modulation of multiple parameters. Finally, we calculate the sensitivity of the absorber and find that it exhibits a high sensitivity of up to 4508.75 GHZ/RIU. Compared to previous studies, our absorber demonstrates superior application value. We believe that our absorber has significant potential to contribute to high-sensitivity sensors, photoelectric detection, photoelectric communication, and other related fields.

## Figures and Tables

**Table 1:** The parameter values of the absorption device.

Parmeter	L_1_	L_2_	R	P	H_1_	H_2_	Graphene Level
**Value (μm)**	3	1.2	4.7	5	0.3	5	0.001

**Table 2:** Comparison of the Absorber in This Paper with Previously Published Absorbers.

Ref.	Materials	Peak Number	Average Absorption	Sensitivity [GHz/RIU]	Q- Factor	FOM (1/RIU)	Tunability
[69]	Au	1	Over 99%	2100	6.8	7.03	Yes
[70]	Graphene	1	99.8%	3923	14.92	6.111	No
[71]	Graphene	3	95.7%	1791	32.354	7.046	No
[72]	BDS	4	99.9%	560	~	~	Yes
[73]	Graphene	13	99.6%	~	~	~	No
[74]	Au	4	98.22%	~	~	~	No
[75]	Graphene	3	99.53%	1387	12.62	1.612	Yes
This work	Graphene	5	98.9%	4508.75	51.32	18.03	Yes

## Data Availability

Publicly available datasets were analyzed in this study. These data can be found here: [https://www.lumerical.com/] (accessed on 1 January 2020).
